# 3D-Printed Temporary Wing Bride as a Temporary Restoration in the Posterior

**DOI:** 10.1155/2024/4869352

**Published:** 2024-05-07

**Authors:** Richard Mosch, Maurice Hatzky, Patricia Hatzky, Constantin von See

**Affiliations:** ^1^Center for Digital Technologies in Dentistry and CAD/CAM, Department of Dentistry, Faculty of Medicine and Dentistry, Danube Private University, Krems an der Donau, Austria; ^2^Department of Prosthodontics and Biomaterials, Faculty of Medicine and Dentistry, Danube Private University, Krems an der Donau, Austria

## Abstract

The fear of a missing tooth often leads to postponing the visit at the dentist. While extraction itself is a major stressful experience for the patient, the presence of visible gaps or missing teeth inside or outside the aesthetic zone is a deal breaker for a lot of patients. Bridging the time spent until inserting any final restoration with a provisional enables the patients to still take part in everyday life. This case report shows a new approach for a fixed dental provisional in the anterior region using a printed wing bridge approach to replace an extracted tooth. The provisional was prefabricated, and extraction and integration of the provisional could be placed in a single visit. The chosen approach shows the integrability of 3D printing in everyday practice providing immediate economical and aesthetic treatment.

## 1. Introduction

Tooth extraction can be a physically and psychologically challenging experience for patients [1]. While various methods of anaesthesia, such as intrasulcular or intraosseous anaesthesia, are available, extractions can still lead to medical emergencies in dental offices [2]. It is therefore essential for dentists and their staff to minimise patient stress during the procedure. Rapid but atraumatic extraction of the entire tooth is required [3], and patients expect gentle and painless treatment as well as immediate restorations, especially in aesthetic areas [4].

Various approaches to temporary restoration can be found in dental practice, with a provisional removable denture supported by the remaining teeth being the most common. However, more comfortable methods such as wing bridges or splints can replace one or two teeth [5, 6]. In addition to the classic function of replacing tooth structure during extraction and protecting it during prosthetic preparations, temporary restorations are becoming increasingly popular with patients for aesthetic reasons [7]. The wing bridge is currently the most effective approach in terms of permanent fixation and a high aesthetic outcome, with various millable and grindable materials available for this purpose [8–10]. Common complications associated with these types of dental bridges are debonding, tooth discoloration, or cavities. Overall, the survival rate is reported to be around 77% in 10 years [11].

Although permanent restorative materials can be used provisionally, their cost-effectiveness must be considered. To address the issues of cost-effectiveness and customisation, 3D-printed temporary restorations can be used instead. The longevity of printed temporaries still needs to be discussed. The ever-improving quality of 3D printers has increased the use and popularity of printable dental restorations, but long-term clinical results are not yet available [12].

Studies have shown the effectiveness of 3D-printed crowns as provisional restorations [13–15] with expanded indications and approvals in recent years to include wing bridges [16].

However, the use of wing bridges has been limited to anterior teeth with sufficient oral bonding surface and exposure to low masticatory forces. This case report presents a new way of restoring a premolar.

## 2. Case Description

**Figure 1:**
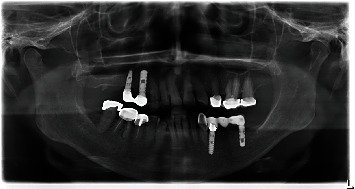
OPTG at first presentation.

In July 2014, the patient sought treatment at the Danube Private University Dental Clinic due to pain in region 46. At the time of the consultation, the patient was classified as ASA class 1 and was not taking any regular medication [17]. At the initial visit, the patient was 69 years of age, and several conservative treatments were performed, including a temporary crown at tooth 45 (Figure 1).

**Figure 2:**
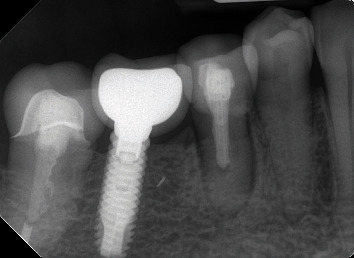
Tooth 45, March 2023.

Tooth 45 remained inconspicuous with the aforementioned restoration until January 2021. However, during a subsequent visit, a carious lesion was observed, and after informing the patient, the crown was removed and the tooth was revised a second time. A Clearfill Core (Kuraray Europe GmbH, Hattersheim, Germany) filling was placed, followed by restoration with a lithium disilicate crown (Emax, Ivoclar Vivadent, Schaan, Liechtenstein). Although no clinical or radiological abnormalities were noted at a follow-up examination one year later, the patient reported mild discomfort in tooth 45. In 2023, the patient presented with a fistulous tract clinically attributed to the same tooth (Figure 2).

The affected area showed visible swelling and mild tenderness at region 45. Based on these observations, the patient was prescribed a 1 g dose of penicillin for antibiotic prophylaxis 24 hours prior to the scheduled extraction in mid-April 2023. The tooth was successfully extracted in its entirety on the scheduled date, but immediate implant placement was not feasible due to insufficient thickness of the buccal lamella and resultant lack of primary stability [18]. After curettage, the wound was closed with a cross suture. The patient had already made the decision to remove the tooth with the desire for a temporary restoration, preferably a fixed one. To achieve this, a digital impression was taken preoperatively using an intraoral camera (Primescan, Dentsply Sirona, Bensheim, Germany).

**Figure 3:**
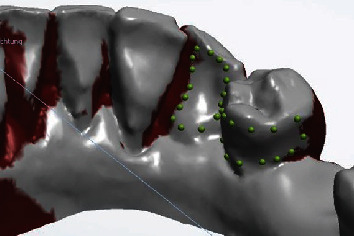
Support surface of the temporary restoration.

**Figure 4:**
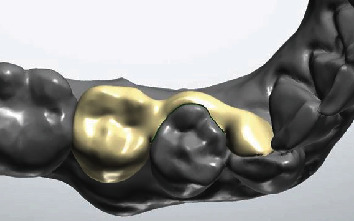
Final design of the wing bridge over 43 and 44 to replace 45.

After exporting the digital impression in the STL format, it was imported into 3Shape Dental System (3Shape, Copenhagen, Denmark), where tooth 45 was digitally removed preoperatively. Teeth 43 and 44 were identified as suitable bridge anchors. To create a printable temporary restoration, a wing bridge was designed on the lingual surface of tooth 43 with an arm extending over tooth 44 along the prosthetic equator (Figures 3 and 4).

**Figure 5:**
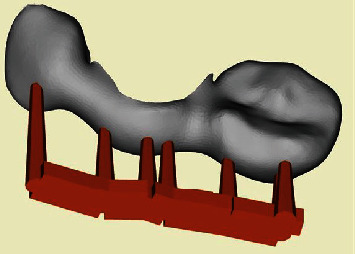
Nesting of the temporary restoration.

**Figure 6:**
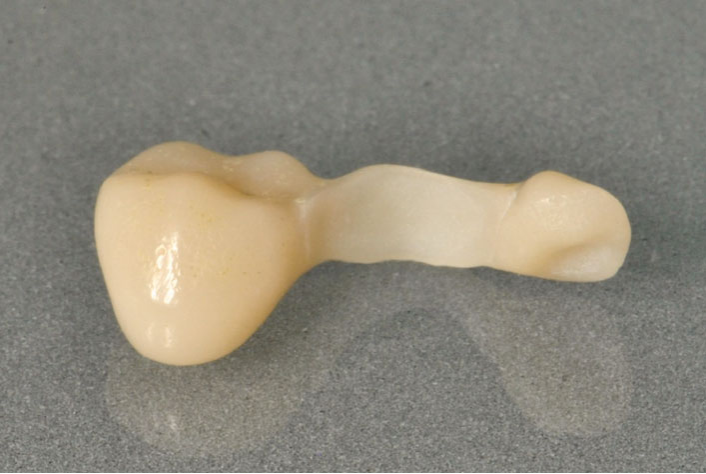
Printed and treated provisional wing bridge.

**Figure 7:**
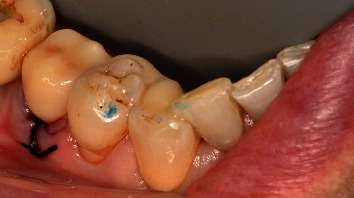
Result of the provisional restoration of tooth 45.

The wing bridge design was exported in STL format and nested using CAMCreator (BEGO, Bremen, Germany) and printed with long time provisional dental resin VarseoSmile Crown Plus (BEGO, Bremen, Germany) (Figure 5). Postprocessing steps were carried out according to the manufacturer's instructions, including an ultrasonic bath with isopropanol and light curing in an Otoflash light oven (BEGO, Bremen, Germany). The cured bridge was sandblasted with Perlablast micro (BEGO, Bremen, Germany) at 1.5 bar using 50 *μ*m glass beads and then polished with pumice stone and polishing compound. It was then stained with Optiglaze color (GC, Tokyo, Japan) (Figure 6). The printed wing bridge was shown to the patient and inserted. Its fit was checked before proceeding with adhesive placement using the acid etching technique and adhesive fixation (RelyX, 3MEspe, Saint Paul, USA). The occlusion was checked and adjusted if necessary before final polishing. The complete restoration was then presented to the patient (Figure 7).

## 3. Discussion

This case shows the successful integration of a 3D-printed temporary wing bride as a provisional restoration.

Patients often request the availability of a fixed temporary restoration to replace a missing tooth. In addition to aesthetics, the provisional should provide functionality, wound protection, stabilisation of adjacent teeth, and maintenance of occlusal harmony.

Immediate implant placement combined with immediate loading is rarely possible due to strict clinical constraints. Sufficient hard tissue without a missing bone wall and a minimal circumferential defect should be available [19]. The absence of a bone wall, particularly the buccal wall, adds an additional aesthetic and functional complexity to immediate implant placement. Aesthetic compromise in the form of reduced alveolar ridge or papillary malformation is possible.

Immediate loading requires additional considerations. The clinical success of the technique depends on several factors: patient selection, bone quality/quantity, number/design of implants, primary stability, occlusal loading, and surgical skill. Of these, primary stability for immediate loading is paramount [20].

Mechanical stability can be achieved while preserving healthy adjacent tooth structure and adjacent soft tissues [21, 22]. In addition to providing a functional replacement, the temporary restoration also fulfils an aesthetic function in the area [23]. The use of a custom-made provisional restoration can protect the soft tissue and prepare it for future procedures, avoiding damage due to unfavourable pressure distribution or swaying of a clasp-supported provisional prosthesis.

Simultaneous implantation with an appropriately designed ovate pontic, an egg-shaped gingival support for the bridge, can positively influence long-term bone levels and achieve better aesthetic results [24]. This allows for a better aesthetic gingival margin with higher patient acceptance. In addition, the oval pontic provides constant pressure for wound closure and prepares the region for papilla formation during wound healing [25].

A single-wing Maryland bridge is recommended over multiple-wing bridges for higher survival rates [26]. Although the cantilevering of the abutment and the consequent blocking of two or more teeth has not been fully investigated in the literature, this is less important in temporary cases than in definitive restorations. In this case, immediate implant placement was not performed [27], but an ongoing shaping of the papilla was performed, taking advantage of the oval pontic.

With the integration of 3D printing into dentistry, 3D-printed temporaries have become another technical option for fixed temporary restorations as a treatment alternative to removable temporaries.

In order for dentists to offer same-day restorations, they need the necessary equipment, such as a 3D printer, software, ultrasonic isopropanol bath, light furnace, and instruments, as well as the technical know-how [28].

However, the implementation of this equipment and knowledge comes at a cost. Despite the initial cost, additive manufacturing of materials has the advantage of being more cost effective than subtractive or directly manufactured temporaries [29]. This can be passed on to the patient, reducing costs for both parties.

Wing bridges as temporary temporaries are an accepted treatment option in place of interim dentures [30]. Fixed dental temporaries require adhesive cementation. If adhesive cementation is used, the tooth structure used must be prepared using 37% phosphoric acid and various monomers depending on the system, with a possible risk of wound infection or allergic reaction [31]. In addition, luting material may be dispersed into the wound [32]. Careful use of adhesives is essential to overcome this potential effect. After cementation and trimming of the bridge, measures to grind the occlusion may be detrimental, as additional rotary grinding may deposit grinding dust in the wound, which would adversely affect wound healing. Cytotoxicity of dental monomers has been reported. Although cytotoxicity induced by dental monomers has been reported, it is too early at this stage to suggest better clinical practices, except perhaps to avoid skin contact with the chosen material [33].

The process of light curing, which releases unbound monomers and is partially associated with cytotoxicity, does not affect wound healing with 3D-printed provisionals. Because the temporary bridge is light cured during the post-treatment process, no additional polymerisation is required except for the cementation process. The soft tissue management provided by this technique allows for stabilisation of the gingiva and bone around the implant site [34].

In addition to functionality, aesthetics is an important consideration for patients when choosing a temporary restoration. Adhesive cementation eliminates the need for additional retention elements such as clasps or similar attachments, which can compromise aesthetics [35]. In addition, as mentioned earlier, the restoration can be personalised for the individual patient using appropriate staining techniques.

## 4. Conclusion

In summary, establishing an in-house provisional restoration workflow is a viable option for dental practitioners. Despite its inherent limitations, this approach allows the provision of fixed provisional solutions for patients who are averse to removable dentures. Particularly in the anterior region, the use of Maryland bridges is an advantageous way of achieving both aesthetic enhancement and soft and hard tissue preservation. This approach offers both patients and clinicians a streamlined, efficient, and economically viable treatment modality.

## Data Availability

The data is available from the corresponding author.
